# Epidemiology, Clinical Spectrum, and Serotype Distribution of NS1-Confirmed Dengue Cases in Andhra Pradesh, India: A Decade-Long Retrospective Study

**DOI:** 10.7759/cureus.108283

**Published:** 2026-05-05

**Authors:** Kodavala Sireesha, Anju Verma, Usha Kalawat, Soumya Dakshinamurthy, Nukaboina Ramakrishna, Deepika Gopi, Pamireddy Madhavi Latha, Alladi Mohan

**Affiliations:** 1 Department of Microbiology, Sri Venkateswara Institute of Medical Sciences, Tirupati, IND; 2 Department of General Medicine, Sri Venkateswara Institute of Medical Sciences, Tirupati, IND

**Keywords:** dengue, epidemiology, neurological manifestations, ns1 antigen, seasonal trends, serotyping, who

## Abstract

Background: Dengue is a major public health concern in India, showing marked seasonal variation and diverse clinical manifestations. Long-term regional data are essential for improving early diagnosis and guiding public health interventions. This study assessed decade-long epidemiological trends, clinical spectrum, and dengue virus serotype distribution among NS1-confirmed dengue cases in Andhra Pradesh, India.

Methods: We conducted a retrospective observational study of 30,086 clinically suspected dengue cases tested for dengue NS1 antigen at a tertiary-care center in Andhra Pradesh, India, between January 2015 and December 2024. Demographic characteristics, urban-rural residence, temporal trends, clinical manifestations classified according to the WHO 2009 dengue classification, and dengue virus serotypes were analyzed. Associations were assessed using chi-square tests, and selected comparisons of clinical manifestation categories were performed using proportion-based analyses.

Results: Among 30,086 suspected cases, 3,899 (13.0%) were NS1-positive. Positivity was higher among males (2,142; 13.6%), children aged ≤18 years (2,235; 20.7%), and rural residents (1,679; 14.7%). Dengue transmission showed consistent post-monsoon peaks from August to October, with a marked decline in 2020 during COVID-19-related movement restrictions. Severe dengue with hemorrhagic manifestations was the most common clinical category (1,734; 44.47%), followed by systemic manifestations (727; 18.64%), neurological involvement (690; 17.68%), gastrointestinal warning signs (307; 7.87%), and hepatic involvement (160; 4.10%). Neurological manifestations were significantly more frequent than gastrointestinal or hepatic involvement. Serotyping of 300 NS1-positive samples showed predominance of dengue virus serotype 3 (111; 37.0%), followed by serotype 2 (70; 23.3%), with mixed serotype infections (28; 9.3%).

Conclusion: This decade-long analysis highlights pronounced seasonality, a substantial pediatric burden, rural predominance, evolving dengue virus serotype circulation, and a notable contribution of neurological manifestations to dengue morbidity in southern India. Strengthened pre-monsoon vector control, early syndromic recognition particularly of neurological involvement, and continued molecular serotype surveillance are essential to reduce dengue-related morbidity in endemic regions.

## Introduction

Dengue fever is a rapidly emerging arboviral infection and a major public health challenge in tropical and subtropical regions [1,2]. It is primarily transmitted by *Aedes aegypti* and *Aedes albopictus* mosquitoes [1]. Over recent decades, rapid urbanization, climate variability, shifting vector distributions, and increased human mobility have substantially expanded dengue transmission [1]. Globally, an estimated 390 million infections occur annually, of which nearly 100 million are clinically apparent, ranging from mild febrile illness to severe dengue characterized by plasma leakage, hemorrhage, organ dysfunction, and shock [1,3].

In India, these global drivers have contributed to a substantial and growing dengue burden, with recurrent outbreaks reported across multiple states [2,4,5]. Transmission typically peaks during the post-monsoon period, reflecting favorable breeding conditions for *Aedes* mosquitoes [2]. Despite ongoing vector control efforts and strengthened surveillance systems, dengue in India continues to exhibit marked interannual variability, periodic outbreaks, and evolving clinical presentations [4,5]. This variability underscores the need for robust, long-term epidemiological data to better understand disease patterns.

Early diagnosis plays a critical role in dengue management, enabling timely identification of warning signs and prevention of progression to severe disease. Detection of the non-structural protein 1 (NS1) antigen during the early febrile phase often precedes IgM seroconversion, making NS1 testing a valuable tool for early case confirmation [5]. Given that NS1 positivity rates, clinical features, and seasonal trends may vary across regions and over time, standardized long-term NS1-based surveillance can provide important insights into dengue epidemiology and support public health planning.

Although several studies from India have examined dengue epidemiology, few have evaluated decade-long trends using standardized NS1 testing in large tertiary-care settings [4,5]. In addition, previously reported age and sex-specific patterns remain inconsistent, with some studies indicating higher positivity among females [6], others showing male predominance [7], and several reporting no significant difference [8]. Age-related trends also vary, with some studies suggesting higher seropositivity in children followed by adults and the elderly [9,10]. Furthermore, while neurological manifestations such as encephalitis, seizures, irritability, and altered sensorium are increasingly recognized, they remain under-reported and insufficiently characterized in large datasets [11]. These inconsistencies highlight the importance of large, standardized datasets to generate more reliable and generalizable evidence.

In this context, the present study analyzes 30,086 suspected dengue cases over a 10-year period (2015-2024) in Andhra Pradesh, India. The study aims to evaluate NS1 antigen positivity trends by age, sex, and residence; characterize seasonal and epidemiological patterns; and describe a broad spectrum of clinical manifestations, including hemorrhagic, systemic, neurological, gastrointestinal, and hepatic features. These findings provide valuable long-term insights to support clinicians, epidemiologists, and public health authorities in improving dengue prevention, early diagnosis, and management strategies.

## Materials and methods

Study design and setting

A retrospective observational study was conducted to analyze clinical and laboratory records of patients presenting with suspected dengue infection between January 2015 and December 2024. Blood samples were collected from inpatients (IP) and outpatients (OP) attending the Department of Medicine, Sri Venkateswara Institute of Medical Sciences (SVIMS), Tirupati, a tertiary-care referral center serving both urban and rural populations across southern Andhra Pradesh, as well as from samples referred by nearby primary health centers and private healthcare facilities. The SVIMS Microbiology Laboratory performed all diagnostic testing. The Institutional Ethics Committee approved the study protocol (IEC No. 1460) and waived informed consent due to the retrospective design and anonymized dataset.

Study population

A total of 30,086 patients underwent dengue NS1 antigen testing during the study period. The cohort comprised 15,769 males (52.4%) and 14,317 females (47.6%). Patients with incomplete demographic information or missing NS1 antigen results were excluded. Age was stratified into three groups: children (≤18 years), adults (18-59 years), and the elderly (≥60 years).

Sample collection and dengue NS1 enzyme-linked immunosorbent assay (ELISA)

Approximately 2-3 mL of venous blood was collected aseptically from each patient. Serum was separated and transferred into sterile, labelled microtubes. Dengue NS1 antigen detection was performed using the Panbio® Dengue Early ELISA kit (Alere Inc., Waltham, MA, USA), which was used consistently throughout the study to ensure methodological uniformity. Samples were stored at -80 °C when not processed immediately [9], and typically completed testing within 48 hours of sample collection. The NS1 antigen detection is most sensitive during the early febrile phase, usually within the first 4-5 days of illness.

Dengue serotyping by real-time reverse transcription polymerase chain reaction (rRT-PCR)

Serotyping was performed on a subset of NS1-positive samples based on sample availability and RNA integrity. Viral RNA was extracted from 140 µL of NS1 antigen-positive serum using the QIAamp Viral RNA Mini Kit (Qiagen, Hilden, Germany) according to the manufacturer’s instructions and eluted in 60 µL of Buffer AVE. The extracted RNA was reverse-transcribed and then analyzed by rRT-PCR using the RealStar® Dengue RT-PCR Kit 1.0 (altona Diagnostics GmbH, Hamburg, Germany) to differentiate dengue virus serotypes 1-4 [12]. Amplication was carried out on the QuantStudio™ 5 Real-Time PCR System (Thermo Fisher Scientific, Waltham, MA, USA) with the following cycling conditions of 20 min at 55 °C, followed by 2 min at 95 °C, and 45 cycles of 15 s at 95 °C, 45 s at 55 °C, and 15 s at 72 °C [12].

Data collection

Demographic and clinical data were retrospectively retrieved from hospital records using a structured data collection proforma [9]. Variables included age, sex, place of residence (urban or rural), month and year of hospital attendance, dengue NS1 antigen test results, and documented clinical manifestations. Clinical information obtained from outpatient and inpatient records was classified in accordance with the WHO-adapted 2009 dengue classification criteria.

Case definitions and symptom categories

Clinical manifestations were analyzed among laboratory-confirmed dengue cases, defined by a positive dengue NS1 antigen test. Clinical features were classified using a WHO-adapted World Health Organization 2009 dengue classification, which includes dengue without warning signs, dengue with warning signs, and severe dengue. For analytical clarity, clinical manifestations were further described as subgroups within these WHO categories. Dengue without warning signs included systemic/constitutional manifestations such as fever with rigors, headache, myalgia, arthralgia, malaise, and retro-orbital pain. Dengue with warning signs included abdominal pain, persistent vomiting, and diarrhea. Severe dengue included severe bleeding, neurological involvement (encephalopathy/encephalitis, seizures, altered sensorium, irritability, somnolence, rigidity), and hepatic involvement (jaundice or severe liver dysfunction) [13]. Percentages for individual symptoms were calculated as proportions within each clinical category, while category prevalence represented the proportion of NS1-positive cases exhibiting at least one manifestation within that category.

Statistical analysis

Demographic characteristics, dengue NS1 antigen test outcomes, clinical manifestations, temporal trends, and serotype distribution were summarized using descriptive statistics. Categorical variables were presented as frequencies and proportions with 95% confidence intervals (CIs), assuming a binomial distribution. Associations between NS1 positivity and demographic variables (age group, gender, residence) were assessed using the chi-square (χ²) test, excluding equivocal results.

Clinical manifestations among NS1-positive cases were classified per the WHO 2009 dengue categories. Prevalence was defined as the proportion of patients with at least one manifestation per category. As categories were not mutually exclusive, patients could contribute to multiple categories; comparisons were therefore descriptive.

Seasonal and temporal trends were assessed using monthly and annual distributions of NS1-positive cases. Serotype distribution was summarized descriptively for samples analyzed by rRT-PCR. A p-value < 0.05 was considered statistically significant. Analyses were performed using JASP version 0.18.1 (University of Amsterdam, Amsterdam, Netherlands) [14].

## Results

Dengue NS1 antigen positivity and demographic profile

A total of 30,086 clinically suspected dengue cases were tested for NS1 antigen between 2015 and 2024, with an overall positivity rate of 13.0%. Age-wise distribution showed the highest positivity among children (≤18 years) and the lowest among the elderly (≥60 years), with statistically significant differences across age groups. A modest male predominance was observed. Rural populations demonstrated significantly higher NS1 positivity compared to urban populations. Detailed demographic distributions and statistical associations are presented in Table 1.

**Table 1:** Age-, gender-, and residence-wise distribution of NS1 antigen test outcomes in the study population (N = 30,086) Data are presented as the number of cases (n) and percentages (%). Statistical significance was assessed using the chi-square (χ²) test. A p-value < 0.05 was considered statistically significant and is indicated by an asterisk (*). χ² values, degrees of freedom (df), and p-values indicate the strength and significance of the association between each demographic variable and NS1 antigen outcome. Positive: NS1 antigen detected, Negative: NS1 antigen not detected, Equivocal: inconclusive NS1 antigen result.

Variables	Number of cases (n)	Positive, n (%)	Negative, n (%)	Equivocal, n (%)	χ²	Degrees of freedom (df)	p-value
Age group (years)							
≤18	10,802	2,235 (20.7)	8,207 (76.0)	360 (3.3)	958.7	4	<0.001*
18-59	14,754	1,450 (9.8)	12,776 (86.6)	528 (3.6)			
≥60	4,530	214 (4.7)	4,183 (92.3)	133 (2.9)			
Gender							
Male	15,769	2,142 (13.6)	13,094 (83.0)	533 (3.4)	11.6	2	0.003*
Female	14,317	1,757 (12.3)	12,072 (84.3)	488 (3.4)			
Total	30,086	3,899 (13.0)	25,166 (83.6)	1,021 (3.4)			
Residence							
Urban	18,672	2,220 (11.9)	15,780 (84.5)	672 (3.6)	41.9	2	<0.00001*
Rural	11,414	1,679 (14.7)	9,386 (82.2)	349 (3.1)			
Total	30,086	3,899 (13.0)	25,166 (83.6)	1021 (3.4)			

Age- and gender-stratified urban-rural analysis of NS1 antigen outcomes

Age- and gender-stratified analysis revealed higher NS1 antigen positivity in rural populations compared to urban populations across most subgroups (Table 2). Among children (≤18 years), positivity was significantly higher in rural females than urban females (22.7% vs 18.2%), whereas no significant difference was observed among males. In adults aged 18-59 years, both males (11.7% vs 10.0%) and females (11.0% vs 7.7%) in rural areas showed significantly higher positivity compared to their urban counterparts. Among the elderly (≥60 years), rural populations had slightly higher positivity than urban populations in both males and females; however, these differences were not statistically significant. Overall, NS1 antigen positivity was higher in rural than urban populations (14.7% vs 11.9%), indicating consistent urban-rural variation across the study population (Tables 1, 2).

**Table 2:** Age- and gender-stratified urban-rural comparison of NS1 antigen test outcomes in the study population (N = 30,086) Values are presented as numbers (n) and percentages (%), with percentages calculated row-wise. χ² values were calculated using the two-by-two Pearson’s chi-square test comparing urban vs rural residence, excluding equivocal results. An asterisk (*) indicates statistical significance (p < 0.05), and rows in bold highlight significant comparisons. Totals reflect all genders combined for each age group and overall.

Age group (years)	Gender	Residence	No. of cases	Positive, n (%)	Negative, n (%)	Equivocal, n (%)	χ² (urban vs rural)	p-value
≤18	Male	Urban	3,260	665 (20.4)	2,493 (76.5)	102 (3.1)	3.39	0.065
		Rural	2,156	504 (23.4)	1,595 (74.0)	57 (2.6)		
	Female	Urban	3,439	625 (18.2)	2,641 (76.8)	173 (5.0)	6.73	0.009*
		Rural	1,947	441 (22.7)	1,478 (75.9)	28 (1.4)		
Total (≤18)			10,802	2,235 (20.7)	8,207 (76.0)	360 (3.3)		
18-59	Male	Urban	4,735	473 (10.0)	4,093 (86.4)	169 (3.6)	7.32	0.007*
		Rural	2,998	351 (11.7)	2,537 (84.6)	110 (3.7)		
	Female	Urban	4,485	347 (7.7)	3,961 (88.3)	177 (3.9)	12.88	<0.001*
		Rural	2,536	279 (11.0)	2,185 (86.2)	72 (2.8)		
Total (18-59)			14,754	1,450 (9.8)	12,776 (86.6)	528 (3.6)		
≥60	Male	Urban	1,507	79 (5.2)	1,392 (92.4)	36 (2.4)	0.42	0.52
		Rural	1,113	70 (6.3)	984 (88.4)	59 (5.3)		
	Female	Urban	1,246	31 (2.5)	1,200 (96.3)	15 (1.2)	0.01	0.91
		Rural	664	34 (5.1)	607 (91.4)	23 (3.5)		
Total (≥60)			4,530	214 (4.7)	4,183 (92.3)	133 (2.9)		
Grand total			30,086	3,899 (13.0)	25,166 (83.6)	1,021 (3.4)		

Dengue virus serotype distribution

**Figure 1:**
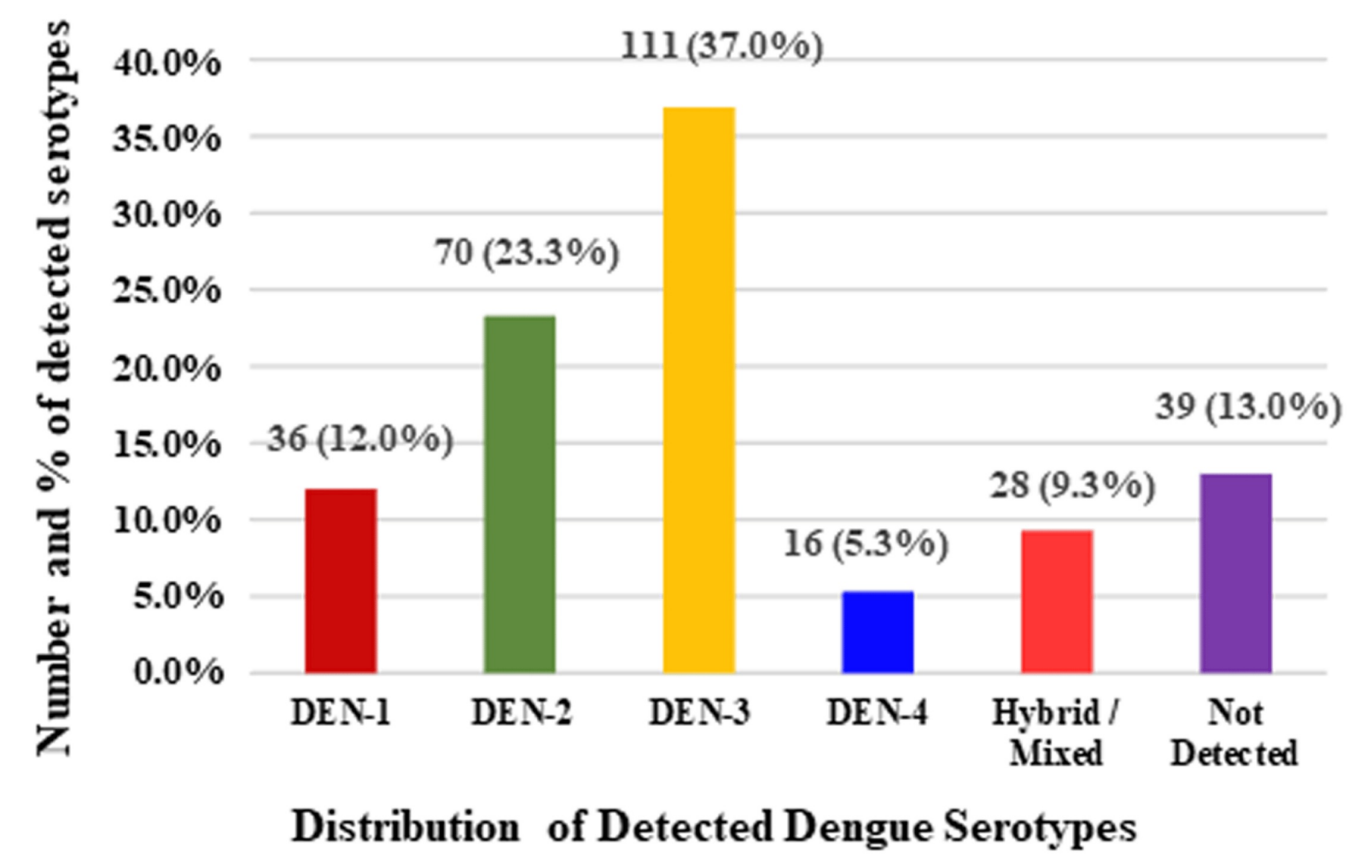
Percentage distribution of detected dengue virus serotypes in the study population (subset of NS1-positive samples, n = 300) DEN-3 predominated (37%), followed by DEN-2 (23%), DEN-1 (12%), and DEN-4 (5%). Mixed serotype infections accounted for 9%, and 13% were undetected for serotype.

Among 300 NS1-positive samples analyzed by rRT-PCR, DEN-3 was the predominant serotype, followed by DEN-2, DEN-1, and DEN-4. Mixed infections accounted for a smaller proportion, while a subset of samples remained undetected despite NS1 positivity (Figure 1).

Temporal and seasonal trends

**Figure 2:**
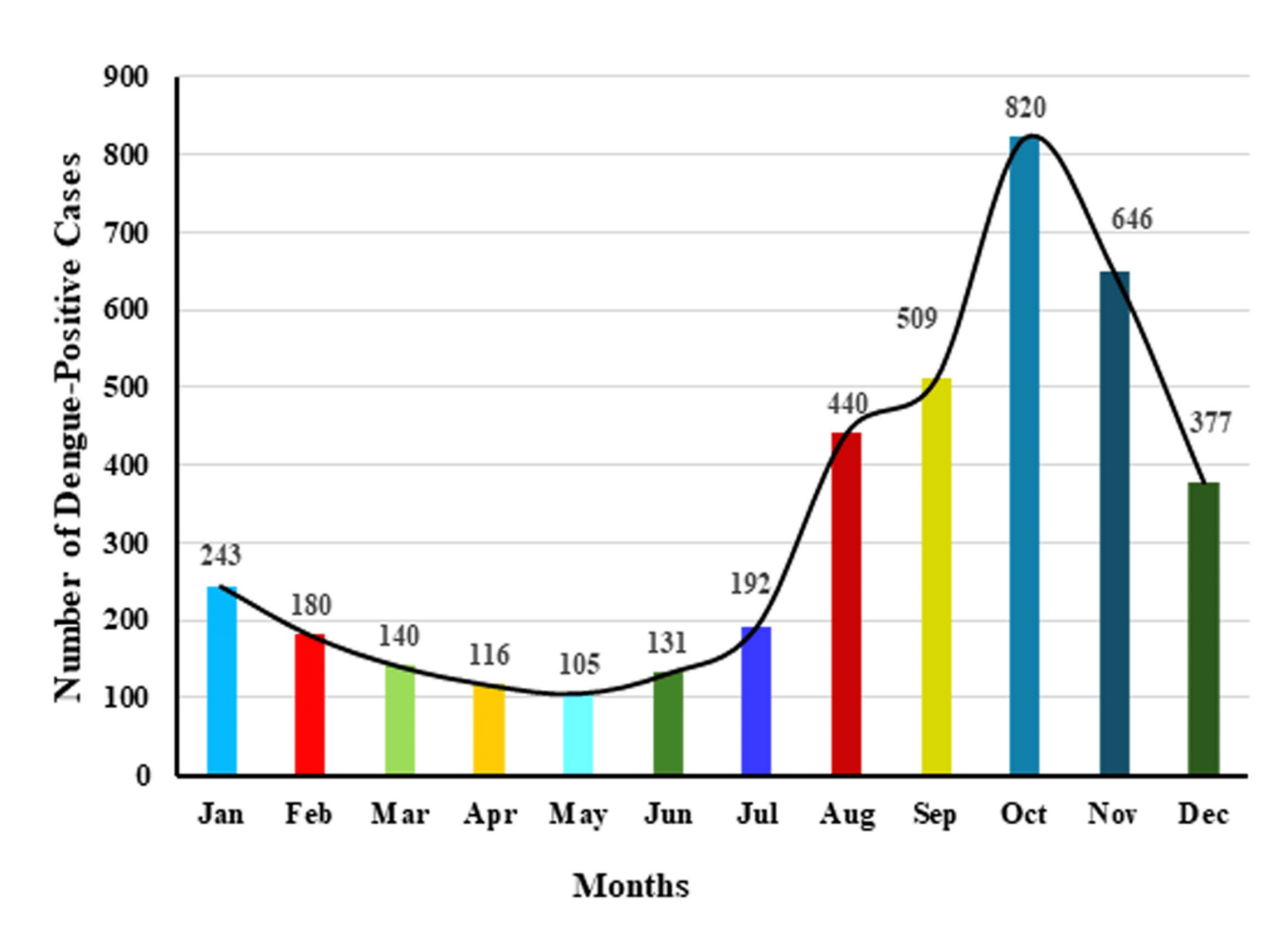
Cumulative monthly distribution of dengue-positive cases (2015-2024) showing seasonal trends and annual peaks Cumulative monthly distribution showing consistent low transmission from January to June, a rise in July, and peak cases from August to October. Case numbers declined steadily in November-December, highlighting recurrent post-monsoon seasonality.

The monthly distribution of dengue cases over the 10-year period (2015-2024) demonstrated a consistent seasonal pattern. Low transmission was observed from January to June, followed by a gradual increase in July and a sharp surge from August to October, reaching a cumulative peak of 820 cases during the post-monsoon period (Table 3; Figure 2). Case numbers then declined steadily in November and December.

**Table 3:** Monthly distribution of dengue cases and NS1 antigen positivity rates in the study population (2015-2024) "Total" indicates the number of cases tested, "positive" indicates NS1 antigen-confirmed dengue cases, and "% positive" indicates the proportion of tested cases that were NS1-positive. NS1 positivity reflects active dengue infection and highlights seasonal and yearly trends in dengue incidence.

Month	2015			2016			2017			2018			2019			2020			2021			2022			2023			2024		
	Total	Positive	% positive	Total	Positive	% positive	Total	Positive	% positive	Total	Positive	% positive	Total	Positive	% positive	Total	Positive	% positive	Total	Positive	% positive	Total	Positive	% positive	Total	Positive	% positive	Total	Positive	% positive
January	291	15	5.2	134	8	6	56	4	7.1	222	18	8.1	174	21	12.1	334	48	14.4	145	7	4.8	357	34	9.5	543	29	5.3	583	59	10.1
February	219	7	3.2	86	5	5.8	60	8	13.3	175	8	4.6	151	26	17.2	187	28	15	140	5	3.6	321	28	8.7	532	23	4.3	474	42	8.9
March	100	2	2	68	2	2.9	59	12	20.3	139	6	4.3	186	28	15.1	88	15	17	126	2	1.6	262	32	12.2	413	15	3.6	397	26	6.5
April	50	3	6	62	3	4.8	73	10	13.7	83	2	2.4	201	47	23.4	13	1	7.7	33	0	0	201	22	10.9	296	15	5.1	246	13	5.3
May	106	5	4.7	55	1	1.8	80	8	10	123	18	14.6	171	24	14	16	2	12.5	10	0	0	181	9	5	258	29	11.2	250	9	3.6
June	125	7	5.6	60	6	10	106	15	14.2	105	4	3.8	186	27	14.5	31	1	3.2	36	4	11.1	145	4	2.8	244	33	13.5	316	30	9.5
July	53	2	3.8	139	1	0.7	200	36	18	154	8	5.2	279	52	18.6	16	0	0	130	5	3.8	268	28	10.4	283	35	12.4	376	25	6.6
August	41	10	24.4	115	10	8.7	281	73	26	136	10	7.4	449	157	35	13	1	7.7	177	16	9	546	44	8.1	433	51	11.8	631	68	10.8
September	118	19	16.1	120	9	7.5	347	72	20.7	162	20	12.3	740	195	26.4	21	5	23.8	298	27	9.1	552	68	12.3	511	51	10	258	43	16.7
October	305	50	16.4	124	11	8.9	431	100	23.2	267	22	8.2	1,237	360	29.1	50	6	12	381	49	12.9	499	102	20.4	716	92	12.8	225	28	12.4
November	249	58	23.3	120	2	1.7	433	105	24.2	239	17	7.1	1,072	287	26.8	82	7	8.5	463	51	11	451	55	12.2	499	43	8.6	227	21	9.3
December	254	52	20.5	98	7	7.1	301	44	14.6	268	24	9	488	94	19.3	134	5	3.7	535	79	14.8	414	25	6	539	39	7.2	254	8	3.1

The highest single-month positivity was recorded in August 2019 (157/449; 35.0%). A marked decline in cases occurred in 2020, coinciding with COVID-19-related movement restrictions, followed by a gradual increase from 2021 onwards.

Analysis across individual months revealed that NS1 positivity rates varied substantially, with low positivity (<10%) in early months (January-June) and higher positivity (up to 35%) during post-monsoon peaks. For example, in 2019, NS1 positivity increased from 12/174 (12.1%) in January to 157/449 (35.0%) in August and 360/1,237 (29.1%) in October. In 2020, case numbers dropped sharply, with only 1/13; 7.7% positive in August, reflecting the impact of movement restrictions.

Cumulatively, these data highlight a recurrent post-monsoon seasonality in dengue transmission, with August-October consistently showing the highest NS1 antigen positivity, followed by a decline towards the end of the year.

Clinical manifestations

Among NS1-positive dengue patients (n = 3,899), severe dengue with hemorrhagic manifestations was the most frequent clinical category (1,734; 44.47%, 95% CI: 42.91-46.03), followed by systemic manifestations (727; 18.64%), neurological involvement (690; 17.68%), gastrointestinal warning signs (307; 7.87%), and hepatic involvement (160; 4.10%) (Table 4).

**Table 4:** Prevalence of clinical manifestations among dengue patients according to clinical categories in the study population (n = 3,899) Prevalence estimates are shown with 95% CI, calculated assuming a binomial distribution with n = 3,899.

WHO-adapted category	Clinical manifestation	Number of cases (n)	Prevalence (%)	95% CI
Dengue with hemorrhagic manifestations	Severe dengue with hemorrhage	1,734	44.47	42.91-46.03
Dengue without warning signs: systemic/constitutional manifestations	Fever with rigors	933	23.93	22.59-25.27
	Headache	907	23.26	21.93-24.59
	Myalgia	948	24.31	22.96-25.66
	Retro-orbital pain	119	3.05	2.51-3.59
Category prevalence		727	18.64	
Severe dengue: neurological involvement	Encephalopathy/ encephalitis	1,406	36.06	34.55-37.57
	Irritability	671	17.21	16.03-18.39
	Somnolence	258	6.62	5.84-7.40
	Seizures	514	13.18	12.12-14.24
	Rigidity	433	11.11	10.12-12.10
	Altered sensorium	855	21.93	20.64-23.22
Category prevalence		690	17.68	
Dengue with warning signs: gastrointestinal manifestations	Diarrhea	374	9.59	8.66-10.52
	Vomiting (persistent)	141	3.62	3.03_4.21
	Gastrointestinal discomfort/pain	405	10.39	9.43-11.35
Category prevalence		307	7.87	
Severe dengue: hepatic involvement	Jaundice	216	5.54	4.83-6.25
	Severe hepatic involvement	104	2.67	2.17-3.17
Category prevalence		160	4.10	

Within systemic manifestations, myalgia (948; 24.31%, 95% CI: 22.96-25.66) and fever with rigors (933; 23.93%, 95% CI: 22.59-25.27) were most commonly observed. Within systemic (constitutional) manifestations, myalgia and fever with rigors were the most commonly observed features, followed by headache. Neurological manifestations were dominated by encephalopathy/encephalitis, followed by altered sensorium and irritability. Gastrointestinal involvement included diarrhea, persistent vomiting, and abdominal discomfort/pain. Hepatic manifestations were the least frequent, comprising jaundice and severe hepatic involvement (Table 4).

Neurological manifestations were more common than gastrointestinal or hepatic involvement, followed by a lower frequency of gastrointestinal compared to hepatic involvement (Table 5). While hemorrhagic manifestations represented the dominant clinical presentation, neurological involvement contributed substantially to dengue-associated morbidity in this cohort.

**Table 5:** Descriptive comparison of major clinical manifestation categories among NS1-positive dengue cases Absolute differences (%) represent percentage-point differences in category-level prevalence. Clinical categories are not mutually exclusive, and patients may be included in more than one category. These comparisons illustrate relative clinical burden and are descriptive in nature; they are not intended to indicate statistical significance. The interpretation column indicates the more prominent manifestation in each comparison.

Comparison	Absolute difference (%)	Interpretation
Neurological vs gastrointestinal (GI)	9.81	Neurological ≫ GI
Neurological vs hepatic	13.58	Neurological ≫ hepatic
Gastrointestinal vs hepatic	3.77	GI ≫ hepatic

Patient information, including demographic characteristics, clinical manifestations, system involvement, and outcomes, was systematically recorded using a structured data collection proforma specifically developed for this study (Appendix 1). 

## Discussion

This 10-year hospital-based study provides comprehensive insights into the epidemiological and clinical patterns of dengue infection in Andhra Pradesh, reflecting dynamic interactions between the dengue virus, mosquito vectors, host immunity, and environmental factors. The observed variations in NS1 antigen positivity by age, sex, residence, season, serotype distribution, and clinical manifestations are biologically plausible and consistent with patterns reported from other dengue-endemic and epidemic regions [15].

In the present study, NS1 antigen positivity was higher among children. This likely reflects immunological differences associated with primary dengue infection, where higher and more sustained viremia leads to increased circulating NS1 antigen levels and improved diagnostic detection [16]. In contrast, adults are more likely to experience secondary infections, where pre-existing heterologous immunity reduces viral replication and shortens the duration of detectable NS1 antigenemia [17]. Immunosenescence in older individuals may further influence immune response dynamics and disease expression. Consistent with these age-related patterns, similar trends have been reported in epidemic settings where pediatric populations show higher dengue positivity rates due to primary infection predominance [16,18].

In line with sex- and residence-related patterns reported in other endemic settings, we observed a modest male predominance in NS1 positivity along with higher positivity rates among rural residents. Sex-based immunological differences, including stronger antiviral responses in females mediated by estrogen-related pathways and X-linked immune gene expression, may contribute to faster viral clearance [19]. Conversely, testosterone-associated immunomodulation in males may permit higher early viral replication, thereby prolonging NS1 detectability. In addition, behavioral factors such as increased outdoor exposure among males may also contribute to this pattern, although these were not directly assessed in the present study [20].

Similarly, the higher NS1 positivity rates observed in rural populations likely reflect ecological conditions favorable for *Aedes* mosquito breeding, including higher humidity, temperature, and availability of stagnant water in rural and peri-urban settings. These environmental factors enhance vector survival and viral replication. Furthermore, differences in vector control practices, socioeconomic conditions, and healthcare access may also contribute to the increased dengue burden observed in rural areas [21].

Seasonal analysis demonstrated a consistent post-monsoon surge in dengue cases, with peak transmission from August to October. This pattern aligns with established evidence that rainfall, temperature, and humidity enhance vector density and reduce the extrinsic incubation period of dengue virus [9]. A marked reduction in cases during 2020 was also observed, likely due to COVID-19-related movement restrictions and altered healthcare-seeking behavior, as reported in other epidemic regions [22].

Serotype analysis revealed predominance of dengue virus serotype 3, accounting for 37% of the 300 NS1-positive samples analyzed by rRT-PCR, followed by dengue virus serotype 2 and other circulating serotypes. Approximately 13% of samples remained undetected due to insufficient RNA quality or low viral load, which has been acknowledged as a limitation. The co-circulation of multiple serotypes is consistent with patterns observed in epidemic settings and increases the risk of secondary infections and antibody-dependent enhancement, contributing to severe disease manifestations [23].

Neurological manifestations constituted an important component of severe dengue presentations in this cohort, reflecting the neurotropic and neuroinflammatory potential of the dengue virus. Although direct central nervous system invasion was not assessed, previous studies have demonstrated dengue viral RNA and antigens in cerebrospinal fluid and neural tissue [11]. Neurological involvement is likely mediated through immune-driven mechanisms, including cytokine-induced blood-brain barrier disruption and systemic inflammatory responses. These mechanisms may be amplified during secondary infections and circulation of potentially neurovirulent serotypes [24,25]. In the present study, neurological involvement (690/3,899; 17.68%) was higher than gastrointestinal and hepatic involvement, indicating its clinically important contribution to disease burden.

Overall, our findings highlight the complex interplay between host immunity, viral characteristics, vector ecology, and environmental factors in shaping dengue transmission and disease severity. These patterns are consistent with observations from other dengue-endemic and epidemic regions, reinforcing the need for integrated surveillance systems combining epidemiological, clinical, and Virological data for early detection and targeted public health response.

Despite the strengths of this study, including a large sample size and a decade-long observation, certain limitations should be acknowledged. The retrospective, hospital-based design may introduce both selection and reporting biases. Serotyping was performed on only a subset of NS1-positive samples, which limits the generalizability of serotype-specific findings. In addition, follow-up and long-term outcome data were not available, restricting assessment of complications beyond the acute phase. Nevertheless, the study provides valuable insights into regional dengue epidemiology, clinical spectrum, and serotype dynamics.

## Conclusions

This decade-long study highlights the influence of host, environmental, and virological factors on dengue transmission and clinical presentation in Andhra Pradesh. Children and rural populations experienced higher NS1 antigen positivity rates, cases peaked consistently post-monsoon, and neurological manifestations were a significant clinical concern. DEN-3 predominated, with mixed serotype infections indicating potential risk for severe disease. These findings underscore the need for early molecular diagnosis, continuous serotype surveillance, and targeted public health interventions to mitigate dengue-related morbidity.

## Appendices

Appendix 1 

**Table 6:** Structured data collection proforma

Section	Variable/clinical feature	Response/options
Patient and study details	Study title	_____________________________
	Patient ID	_____________________________
	Date of admission	_____________________________
Demographics	Age (years)	_____________________________
	Gender	☐ Male ☐ Female ☐ Other
	Residence	☐ Urban ☐ Rural
	Contact number (optional)	_____________________________
Clinical features	Fever	☐ Yes ☐ No
	Headache	☐ Yes ☐ No
	Myalgia	☐ Yes ☐ No
	Rash	☐ Yes ☐ No
	Nausea/vomiting	☐ Yes ☐ No
	Other symptoms (specify)	☐ Yes ☐ No
Clinical category/system involvement	Severe dengue: hemorrhagic manifestations	☐ Yes ☐ No
	Dengue systemic: fever with rigors	☐ Yes ☐ No
	Dengue systemic: retro-orbital pain	☐ Yes ☐ No
	Severe dengue: encephalopathy	☐ Yes ☐ No
	Severe dengue: irritability	☐ Yes ☐ No
	Severe dengue: somnolence	☐ Yes ☐ No
	Severe dengue: seizures	☐ Yes ☐ No
	Severe dengue: rigidity	☐ Yes ☐ No
	Severe dengue: altered sensorium	☐ Yes ☐ No
	Dengue with warning signs: diarrhea	☐ Yes ☐ No
	Dengue with warning signs: abdominal pain/GI discomfort	☐ Yes ☐ No
	Severe dengue: jaundice	☐ Yes ☐ No
	Severe dengue: hepatic involvement	☐ Yes ☐ No
	Other (specify)	☐ Yes ☐ No
Outcome	Discharged/recovered	☐ Yes ☐ No
	Complications (if any)	☐ Yes ☐ No
	Date of discharge	_____________________________
